# Predicting *in vivo* MRI Gradient-Field Induced Voltage Levels on Implanted Deep Brain Stimulation Systems Using Neural Networks

**DOI:** 10.3389/fnhum.2020.00034

**Published:** 2020-02-20

**Authors:** M. Arcan Erturk, Eric Panken, Mark J. Conroy, Jonathan Edmonson, Jeff Kramer, Jacob Chatterton, S. Riki Banerjee

**Affiliations:** ^1^Restorative Therapies Group, Implantables R&D, Medtronic PLC, Minneapolis, MN, United States; ^2^Cardiac Rhythm Heart Failure, Device Product Engineering, Medtronic PLC, Minneapolis, MN, United States

**Keywords:** MRI gradient-field modeling, gradient-induced voltage, DBS MR conditional testing, DBS implant trajectories, integrating machine learning and computational modeling

## Abstract

**Introduction:**

MRI gradient-fields may induce extrinsic voltage between electrodes and conductive neurostimulator enclosure of implanted deep brain stimulation (DBS) systems, and may cause unintended stimulation and/or malfunction. Electromagnetic (EM) simulations using detailed anatomical human models, therapy implant trajectories, and gradient coil models can be used to calculate clinically relevant induced voltage levels. Incorporating additional anatomical human models into the EM simulation library can help to achieve more clinically relevant and accurate induced voltage levels, however, adding new anatomical human models and developing implant trajectories is time-consuming, expensive and not always feasible.

**Methods:**

MRI gradient-field induced voltage levels are simulated in six adult human anatomical models, along clinically relevant DBS implant trajectories to generate the dataset. Predictive artificial neural network (ANN) regression models are trained on the simulated dataset. Leave-one-out cross validation is performed to assess the performance of ANN regressors and quantify model prediction errors.

**Results:**

More than 180,000 unique gradient-induced voltage levels are simulated. ANN algorithm with two fully connected layers is selected due to its superior generalizability compared to support vector machine and tree-based algorithms in this particular application. The ANN regression model is capable of producing thousands of gradient-induced voltage predictions in less than a second with mean-squared-error less than 200 mV.

**Conclusion:**

We have integrated machine learning (ML) with computational modeling and simulations and developed an accurate predictive model to determine MRI gradient-field induced voltage levels on implanted DBS systems.

## Introduction

More than 60% of patients implanted with a deep brain stimulator (DBS) need an MRI within 10 years after implantation (Falowski et al., 2016). Structural and functional MRI is commonly used to evaluate patients with neurodegenerative diseases, including Parkinson’s disease, essential tremor, and dystonia. Diffusion tensor imaging and functional MRI of patients implanted with DBS systems may enable better understanding of neurogenerative disorder mechanisms (McIntyre et al., 2004; Kohl et al., 2014; Schläpfer and Kayser, 2014; Lipsman and Lozano, 2015; Herrington et al., 2016) and neuromodulation therapies to improve outcomes in existing indications and expand therapies with new indications. DBS systems should be designed to mitigate for unintended interactions between implanted systems and strong electromagnetic (EM) fields produced by MRI scanners, which may otherwise pose significant hazards to patients (Nyenhuis, 2003). Foreseeable potential hazards to patients from MRI EM fields include tissue damage due to heating, vibration, force and torque, unintended tissue stimulation and malfunction of the implanted system. The second edition of the International Standards Organization (ISO) Technical Specification (TS) “Assessment of the safety of magnetic resonance imaging for patients with an active implantable medical device,” governs test methods to evaluate potential hazards (ISO/TS-10974, 2018) due to MRI fields.

Deep brain stimulation systems should also be designed for time-varying MRI gradient-fields, which can induce electric fields (E-fields) and current flow in the human body that may cause unintended tissue stimulation (Ham et al., 1997; Schaefer et al., 2000; Glover, 2009). MRI gradient-field induced E-fields may generate extrinsic voltage potential along implanted elongated conductive structures such as DBS leads/extensions, between electrodes and conductive neurostimulator (device) enclosure. Potential hazards due to MRI gradient-induced extrinsic voltage along DBS systems include unintended tissue stimulation and device malfunction. Test methods to assess these hazards are defined in parts of Clause 13 and 16 of ISO/TS 10974 (ISO/TS-10974, 2018), and to thoroughly assess these hazards, clinically relevant gradient-field induced voltage levels on DBS systems need to be calculated, and used as test exposure levels.

Electromagnetic simulation methods described in Annex A.3.4 for ISO/TS 10974 (ISO/TS-10974, 2018) are commonly used to derive MRI gradient-field induced voltage levels on active implanted medical devices (AIMDs) with leads. First, realistic MRI gradient coil models are generated. Next, using these coil models, E-fields in the tissues of anatomically accurate human body models [e.g., virtual population (Gosselin et al., 2014)] are simulated (Zhao et al., 2002; Bencsik et al., 2007). Then, the tangential component of the E-fields from human simulations are extracted along clinically relevant DBS implant trajectories resulting in one-dimensional E-field exposure distributions. Finally, the tangential E-field exposures are integrated to calculate gradient-field induced voltage levels between electrodes and neurostimulator of the implanted DBS system.

Incorporating additional human body models and therapy implant routing pathways into the EM simulation library can help to achieve more clinically relevant and accurate test exposure levels, however, adding new anatomical human models into the EM simulation library is time-consuming, expensive and not always feasible. In this work, we integrated machine learning (ML) with computational EM modeling and developed a predictive algorithm using artificial neural networks (ANN) to predict *in vivo* MRI gradient-field induced extrinsic voltage levels on implanted DBS systems.

## Background

E-fields are induced inside the human body due to time-varying MRI gradient magnetic fields. In the presence of AIMDs with long conductive components such as DBS leads and extensions, MRI gradient-field induced E-fields can generate extrinsic electric potential between spatially separated electrodes of an AIMD. Gradient-field induced extrinsic electric potential can be calculated by integrating the tangential component of the E-field along trajectory of the implanted system:

$$V_{g\,r\,a\,d\,i\,e\,n\,t}=\int_{0}^{L}\vec{E}\cdot d\,\vec{l}$$

where $\vec{E}$ is the E-field, *L* is the length of the AIMD trajectory and *V*_*gradient*_ is the MRI gradient-field induced extrinsic potential.

In this work, MRI gradient-field induced extrinsic electric potential between DBS therapy electrodes and the implanted conductive neurostimulator are evaluated. Terms induced voltage and voltage levels are used interchangeably to refer to MRI gradient-field induced extrinsic potential between DBS electrodes and neurostimulator.

## Methods

### EM Simulation Methodology and Dataset Generation

**FIGURE 1:**
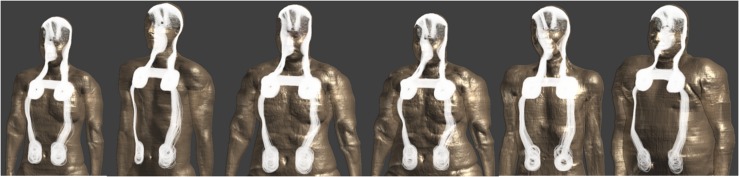
Clinically relevant DBS lead/extension routing trajectories in Virtual Population models are shown.

The Tier 3 EM simulation method described in Annex A.3.4 of ISO/TS 10974 edition 2 (ISO/TS-10974, 2018) is used to calculate *in vivo* gradient-field induced voltage levels on implanted DBS systems (Supplementary Figure S1). The computational modeling and simulation workflow is verified and validated following the principles of the ASME V&V 40 *Assessing Credibility of Computational Modeling through Verification and Validation: Application to Medical Devices* (V&V 40, 2018). Five gradient coil model sets consisting of *X*-, *Y*-, and *Z*-axis coils representing clinical MRI scanners are developed (Supplementary Table S1). Six adult Virtual Population 3.0 models (Gosselin et al., 2014) are used in simulations (Supplementary Table S2). The magneto quasi-static solver of Sim4Life software v3.4 (Zurich MedTech, Zurich, Switzerland) is used to simulate E-field distributions generated in the conductive tissues of the human body, at scan locations spanning human models from head to lower extremities at 10 cm increments, resulting in 1080 total volumetric E-field simulations. 3007 unique clinically relevant DBS implant lead/extension routing trajectories are implemented in six human body models using an in-house developed fully automated software (Figure 1). Tangential E-field distributions along DBS implant trajectories are then extracted and integrated to calculate MRI gradient-field induced voltage values at maximum gradient slew rate of 200 T/m/s. For each unique implant trajectory, gradient coil model set and scan *z*-axis landmark location configuration, final gradient-induced voltage levels are calculated by combining voltage levels due to *X*, *Y*, and *Z*-axis coils in a sum-of-magnitude fashion to conservatively account for all three axis simultaneously slewing at 200 T/m/s. The final gradient-induced voltage dataset is comprised of 180,420 unique induced voltage predictions.

### Feature Selection

Each unique gradient coil, scan landmark location, body model and implant routing configuration produce a different gradient-field induced voltage value. Ten features are derived based on subject, DBS implant and MRI scan related attributes (Table 1). Three binary categorical variables represent DBS implant related features such as brain hemisphere where the lead tip is placed (left or right), side of the neck the DBS extension is tunneled (left or right) and anatomical location where the neurostimulator is implanted (pectoral or abdominal). DBS lead and extension length (in meters) are the other implant-related features. Subject-related features are height (in meters) and mass (in 100 kg). MRI scan related features are scan z-axis isocenter location relative to top of the head, MRI gradient coil diameter and coil length (all in meters).

**TABLE 1:** Ten features are used in predictive models.

**Feature#/ type**	**Feature name**	**Description**	**Units or options**
1/Subject	Height	Height of the subject	In meters
2/Subject	Mass	Mass of the subject	In 100 kg units
3/Implant	Brain	Brain hemisphere where the DBS lead tip is placed	Left or right (Binary)
4/Implant	Neck	Side of the neck the DBS extension is tunneled, and side of the body the DBS neurostimulator is implanted	Left or right (Binary)
5/Implant	INS	DBS neurostimulator implant location	Pectoral or abdominal (Binary)
6/Implant	Lead length	DBS lead length	In meters
7/Implant	Extension length	DBS extension length	In meters
8/MRI scan	LM (landmark)	MRI scan *z*-axis isocenter location relative to top of the head	In meters
9/MRI scan	Coil diameter	MRI gradient coil diameter	In meters
10/MRI scan	Coil length	MRI gradient coil length	In meters

*First two features are subject related, five features are DBS implant related, and remaining three features are MRI gradient coil and scan related.*

It is important to note that implant-related features do not guarantee a unique implant routing trajectory. For instance, there are multiple replicate implant trajectories implemented in the Duke anatomical model that are placed in the left brain hemisphere, tunneled through the left side of the neck with the neurostimulator implanted in the pectoral region, with a 40 cm long DBS lead and a 60 cm long extension (Supplementary Figure S2A). The feature set is selected based on easily identifiable subject and DBS implant-related properties; more advanced features that may enable unique identification of replicate implant trajectories are not considered. Therefore, the selected feature set cannot differentiate between replicate DBS implant trajectories that share same implant-related features.

Gradient-induced voltage levels of replicate DBS implant configurations are averaged to speed up the model training and hyper-parameter tuning process. Averaging of replicate configurations reduced the “Extended-Dataset” size from 180,420 to 10,620 rows in the “Condensed-Dataset.”

### Machine Learning Algorithm Selection and Model Parameter Optimization

Performance of five ML regression algorithms are evaluated on the Condensed-Dataset: support vector regression with linear kernel (SVMLIN), support vector regression with radial basis function kernel (SVMRBF), random forest regressor (RF), gradient boosting regressor (GB), and ANN with two fully connected hidden layers (NN). For all five algorithms, model parameter optimization is performed using “GridSearchCV” from scikit-learn package (Pedregosa et al., 2011). To evaluate generalizability of ML algorithms, training is performed on five body-models excluding “Fats” and model performance are evaluated on the “Fats” induced voltage values. Mass of “Fats” is ∼40 kg higher than the next heaviest body-model, therefore this approach is expected to provide insights on model generalizability by assessing the performance on a “previously unseen” case. *R*-squared (*R*^2^), mean-squared-error (MSE) and median-absolute-error (MAE) metrics are calculated to evaluate the performance of these ML algorithms.

### Neural Network Predictive Model Performance Evaluation

Neural network model training is performed using the Condensed-Dataset and performance metrics are evaluated on the Extended-Dataset. Leave-one-out cross validation is performed by training the NN model on five body-models and evaluating the performance on the body-model that is not included in training to assess model generalizability. Model training history with training and validation losses are evaluated to avoid overfitting. Final NN model with optimized parameters is then trained using the entire Extended-Dataset including all six body-models. Since a separate validation dataset was not available to evaluate the final NN model trained on the entire dataset, most conservative NN model prediction uncertainty levels from the leave-one-out cross validation analysis are used to estimate gradient-induced voltage prediction uncertainty (Abu-Mostafa et al., 2012).

### Software Used

A list of software scripting and analysis tools used in this work are provided in the Supplementary Material.

## Results

### Data Exploration

Three axis sum-of-magnitude gradient induced voltage levels at each principal axis slewing at 200 T/m/s are calculated. Example boxplots shown in Supplementary Figures S3, S4 demonstrate dependence of voltage levels to different features. In general, the abdominal INS implant location has higher induced voltage levels because of the longer straight path of the DBS extension inside the body. Even though there is not a significant difference between DBS leads implanted left or right brain hemisphere, contralateral routing pathways tend to induce slightly higher voltage levels compared to ipsilateral pathways. Induced voltage levels have strong dependence on scan location, with head and lower torso/pelvis scans inducing highest values, whereas chest scans are predicted to have lower induced voltage levels. Mass of the body model is also positively correlated with induced voltage levels. Highest gradient-induced voltage is 5.38 V, and occurred in the “Fats” model for an abdominal implant trajectory. Highest gradient-induced voltage values for other human models ranged between 3.17 and 3.88 V.

As described in the Feature Selection section, selected features cannot uniquely identify DBS implant routing trajectories that are in the Extended-Dataset. For example, replicate routing pathways shown in Supplementary Figure S2A share same feature-set, and generate different induced voltage levels (blue bars in Supplementary Figure S2B). A Condensed-Dataset is generated by averaging induced voltage levels of replicate implant routing pathways (Supplementary Figure S2B: average induced voltage value for the Condensed Dataset: 0.855 V), and this process reduced the size of the dataset from 180,420 to 10,620 rows.

### Algorithm Selection

To evaluate generalizability of ML algorithms, training is performed on five anatomical-models excluding “Fats” and performance are evaluated on the “Fats” induced voltage values. Performance of tuned SVMLIN, SVMRBF, RF, GB, and NN predictive models are shown in Supplementary Figure S5, with red representing the identity line. SVMLIN and SVMRBF models performed poorly. Even though *R*^2^ of RF and GB models are higher than 0.8, both models are biased and consistently underestimate “Fats” induced voltage levels. The NN model has the lowest MSE, highest *R*^2^, does not have a consistent bias, and therefore NN algorithm is selected for this study.

### Neural Network Model Evaluation

Neural network models with two to five fully connected hidden layers are evaluated and no significant performance boost is observed beyond two. Optimal model parameters are found to be a fully connected two layer NN with 25 and 120 units in first and second hidden layers, with no dropout, a batch size of 32 and 300 epochs; using GridSearchCV hyperparameter optimization method. Leaky rectified linear unit (ReLU) activation functions are used in all layers. As described before, all model training has been performed using the Condensed-Dataset. The two-layer ANN predictive models are capable of producing thousands of gradient-induced voltage predictions in less than a second.

Leave-one-out training history plots are shown in Supplementary Figure S6, with red and blue lines representing training and validation loss, respectively. Training loss and validation loss curves follow each other closely in all anatomical models except “Fats,” indicating that training dataset with remaining five anatomical models is not able to fully represent the validation dataset of “Fats” induced voltage levels.

**FIGURE 2:**
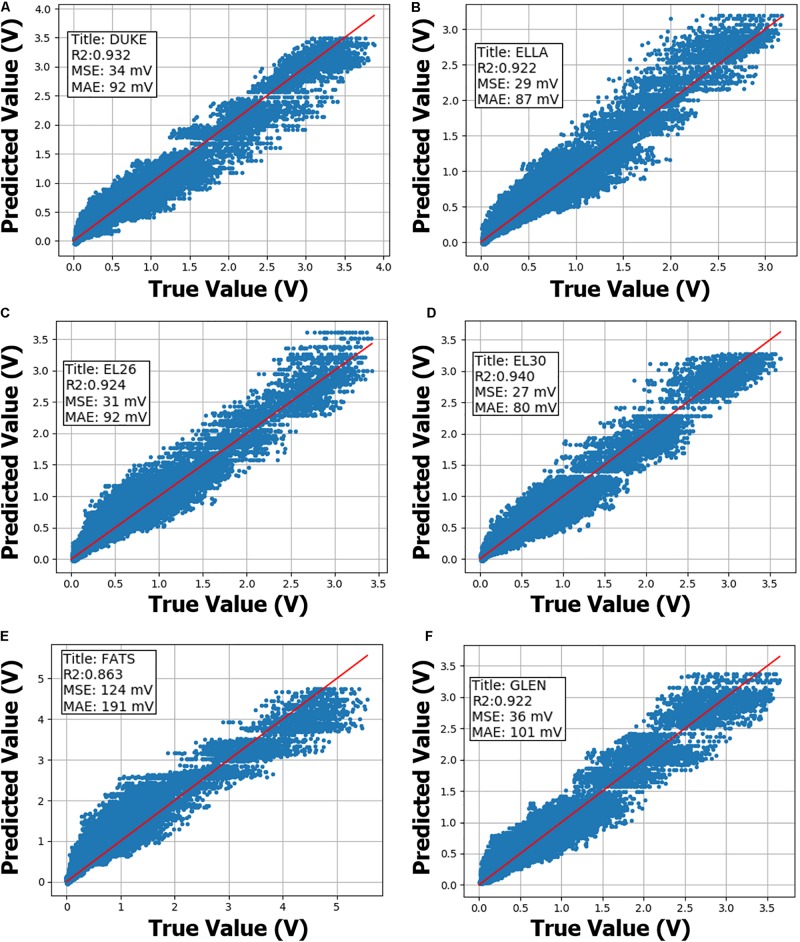
Neural network (NN) leave-one-out cross validation performance on the Extended-Dataset are shown. NN model training is performed on the Condensed-Dataset with the annotated body-model being left out. Sub-figures **(A–F)** summarize cross validation performance using anatomical models Duke, Ella, morphed Ella with BMI of 26, morphed Ella with BMI of 30, Fats and Glenn, respectively, as annotated within. Then, trained NN model performance is evaluated on the annotated body-model from the Extended-Dataset. *R*-squared (*R*^2^), mean-squared-error (MSE) and median-absolute-error (MAE) metrics are also annotated inside the sub-figures. *X*- and *Y*-axis show true and predicted gradient-induced voltage levels in units of V.

Leave-one-out cross validation results on the Extended-Dataset are shown in Figure 2. Extended-Dataset includes all data points with replicate routing pathways and associated variability (Supplementary Figure S2), therefore the Extended-Dataset therefore truly gauges the performance of the predictive models while accurately incorporating the added variability due to replicate routing pathways.

Neural network models on Extended-Dataset exhibited a similar pattern, with induced voltage predictions on “Fats” body model having worse performance metrics (*R*^2^ = 0.86, MSE = 124 mV) compared to other five body models (*R*^2^ > 0.92, MSE < 40 mV). Final NN model prediction uncertainty is derived from the leave-one-out analysis, and most conservative MSE and MAE values of 124 and 191 mV, respectively, are used.

## Discussion

Computational modeling and simulations can be used to generate large synthetic datasets. Incorporating ML with computational modeling and simulations has a potential to provide better understanding of biological and physical phenomena, solve ill-posed inverse problems, optimize complex design problems in a variety of fields (Burrascano et al., 1999; Kim et al., 2007; Tolk, 2015; Hughes et al., 2017; Cohen et al., 2018; Ianni et al., 2018; Pérez et al., 2018; Deist et al., 2019; Kiarashinejad et al., 2019; Meliadò et al., 2019; Tahersima et al., 2019; Vinding et al., 2019). In this work, we have generated a dataset by modeling and simulating MRI gradient-field induced voltage levels on implanted DBS systems, using realistic MRI gradient coil models, six adult anatomical human models (Gosselin et al., 2014) and clinically relevant DBS implant trajectories. We then selected a limited feature set based on the properties of the implanted DBS system, patient, MRI system and scan location, and trained predictive models on the synthetic dataset to predict gradient-field induced voltage levels.

The most dissimilar anatomical model in terms of mass, “Fats,” is used to evaluate the model generalizability performance of five ML algorithms. NN models are found to perform better than other algorithms tested in terms of model generalizability, and are used in this study.

Leave-one-out cross validation results demonstrate that predictive performance deteriorates if the physical characteristics of the anatomical model are not accurately represented in the training set (see Figure 2E, “Fats” cross-validation plot). “Fats” anatomical model has the highest BMI and is ∼40 kg heavier than the next heaviest in the current dataset. A typical DBS implant in the “Fats” model might have a substantially different trajectory compared to other models included in the dataset, therefore exposing the implant to significantly different tangential E-field distributions. Even though predictive performance on the “Fats” induced voltage predictions is worse compared to other anatomical models, MSE, and MAE are less than 200 mV, and is acceptable for this application. If more anatomical models with a larger variety of physical properties are included, predictive performance of the trained ML models are expected to improve. Final NN model was trained using the entire “Extended-Dataset,” and since a separate validation set was not available, prediction uncertainty was derived from the leave-one-out cross-validation analysis (Abu-Mostafa et al., 2012), and is a limitation of this study.

Two-layer ANN predictive model developed in this study can perform thousands of gradient-induced voltage predictions in less than a second. In comparison, Tier 3 EM MRI gradient induced E-field simulations take between 3 and 5 min on a workstation with Intel Xeon E3-1535M CPU (2.9 GHz). Simulating E-field distributions inside a human body model using a set of gradient coils (*X*-, *Y*-, and *Z*-axis) therefore takes at least 9 min. After gradient-induced E-field simulations are completed, extracting tangential component of the E-field along hundreds of DBS implant trajectories and computing the induced voltage take less than a second. In the most conservative scenario, ANN predictive model offers a speed-up of 500-fold (1 s vs. 9 min). On the other hand, Tier 3 EM simulation approach requires availability of anatomical body models and clinically relevant DBS routing pathways. Obtaining, creating, or morphing an anatomical body model can be time-consuming, expensive and may not always be feasible. Implementing a clinically relevant DBS implant trajectory in anatomical body model using an in-house software take between 10 to 100 s depending on the implanted system length, complexity of the DBS trajectory and quality of the anatomical body model tissue segmentation; or several hours if done manually by an experienced design technician. In cases where additional anatomical body models and/or DBS routing pathways are not available, Tier 3 EM simulation approach would require days/weeks to complete, whereas the predictive ANN model doesn’t require availability of additional anatomical body models or DBS routing pathways.

A limitation of this study is that, only conventional symmetric whole-body MRI gradient coil models are evaluated at a maximum slew rate of 200 T/m/s. Gradient coils with higher peak amplitude (G_max_) and peak slew rate are desired to improve image quality and speed for echo planar imaging, diffusion-weighted imaging and several other advanced imaging protocols (Le Bihan et al., 1986; Young et al., 1987; Turner et al., 1990; Wong et al., 1991; Basser and Pierpaoli, 1996), and significant research efforts are being carried out to develop such coils. Local head/neck gradient coils have been evaluated and developed to improve gradient performance compared to whole-body gradient coils (Alsop and Connick, 1996; Crozier et al., 1999; Chronik et al., 2000; Tang et al., 2016). High-performance research systems for imaging human brain connectivity and microstructure have been developed with G_max_ of 300 mT/m (McNab et al., 2013) or slew rate of 1200 T/m/s (Weiger et al., 2018). Local head gradient coils have the potential to reduce E-fields in the body/torso and to elicit peripheral nerve stimulation (PNS) threshold for *in vivo* human imaging (Chronik and Rutt, 2001; Zhang et al., 2003; Tan et al., 2019). Advancements in functionalized anatomical models with nerve trajectories (Lloyd et al., 2018; Neufeld et al., 2018) coupled with EM and neurodynamic simulations (Davids et al., 2017, 2019) have the potential for designing high-performance MRI gradient coils. If higher performance gradient coils and/or local head gradient coils become available in the clinic, such designs need to be incorporated into the MRI gradient-field induced voltage analysis workflow for accurately evaluating the safety of implanted DBS systems.

## Conclusion

We have integrated ML with computational modeling and simulations and trained a neural network to predict MRI gradient-field induced voltage levels on implanted DBS systems. The predictive model is capable of producing thousands of gradient-induced voltage predictions in less than a second (speed-up of > 500-fold compared to EM simulation method) with mean-squared-error less than 200 mV. The predictive model developed in this study can be used to determine clinically relevant MRI gradient-induced voltage exposure levels on implanted DBS systems.

## Data Availability Statement

The datasets for this article are not publicly available because datasets are Medtronic confidential. Requests to access the datasets should be directed to ME, arcan.a.erturk@medtronic.com.

## Author Contributions

ME conceived the study, performed the computational modeling and simulations, trained the machine learning models, and wrote the manuscript. EP helped with machine learning model development and analysis. MC and JE helped with gradient-induced voltage modeling and simulations. EP, MC, JC, and RB helped with the study design. All authors reviewed and edited the manuscript.

## Conflict of Interest

The authors declare that this study received funding from Medtronic. The funder had the following involvement with the study: study design, collection, analysis, interpretation of data, the writing of this article, and the decision to submit it for publication.
