# Supplementary material for: Predicting in vivo MRI Gradient-Field Induced Voltage Levels on Implanted Deep Brain Stimulation Systems Using Neural Networks
Source: Front Hum Neurosci. 2020 Feb 20;14:34. doi: 10.3389/fnhum.2020.00034 (PMC7044348; doi:10.3389/fnhum.2020.00034)
Supplement: Supplementary file 1 [file Data_Sheet_1.docx]

Supplementary Material

# Software Used

- Magneto quasi-static electromagnetic (EM) simulations to compute Electric-field (E-field) distributions inside Virtual Population human body models are performed using Sim4Life version 3.4 (Zurich MedTech, Zurich Switzerland).
- Simulation results are exported to .MAT files using custom scripts developed in Python scripting interface (Python 2.7) within the Sim4Life user interface.
- Clinically-relevant deep brain stimulation (DBS) lead/extension implant trajectories are generated using an in-house developed fully-automated Matlab software (MathWorks, Natick, MA).
- Tangential E-field distributions along DBS implant trajectories are extracted and induced voltage values are calculated using a custom Matlab script.
- Machine learning (ML) algorithms are implemented using Python 3.7. Scikit-learn (0.20.3), Keras (2.2.4) with Tensorflow backend and other Python packages such as Matplotlib, SciPy, pandas, NumPy are used. Model training is performed using a mobile workstation with Intel Xeon E3-1535M CPU (2.9GHz) and 64GB RAM. GPU acceleration is not adopted. Training of NN models take between 1 to 5 minutes.

# Supplementary Figures and Tables

## EM Simulation Methods


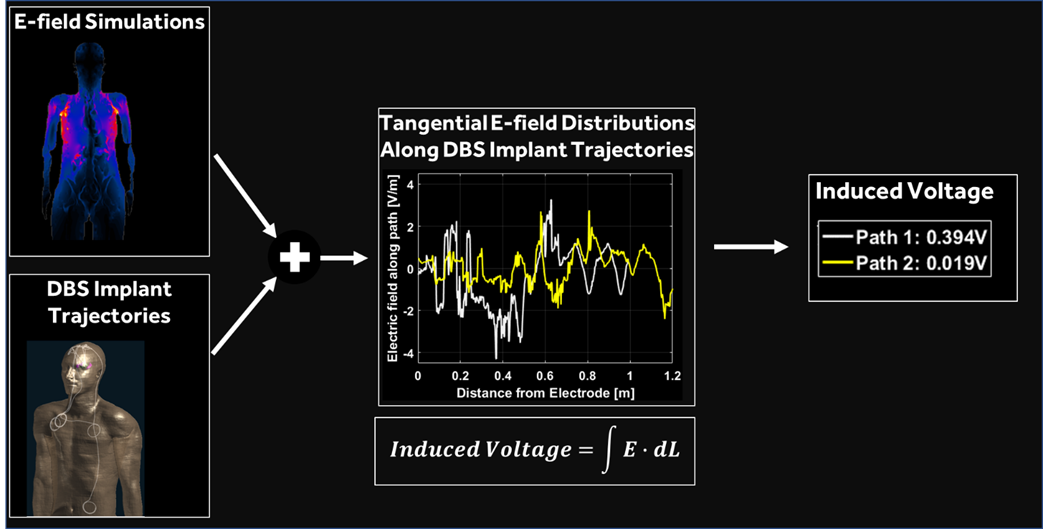


Figure S1. Tier 3 MRI gradient-field induced voltage electromagnetic simulation method (ISO/TS-10974 2018).

**Table S1**. Description of gradient coil models used in simulations.

| **Coil Set #** | **Description** | **Diameter (cm)** | **Length (cm)** |
| --- | --- | --- | --- |
| **1** | Narrow bore gradient coil system representing clinical systems with shorter length static magnet designs. | 60 | 110 |
| **2** | Narrow bore gradient coil system representing clinical systems with average length static magnet designs. | 60 | 150 |
| **3** | Narrow bore gradient coil system representing clinical systems with longer length static magnet designs. | 60 | 190 |
| **4** | Wide bore gradient coil system representing clinical systems with shorter length static magnet designs. | 70 | 110 |
| **5** | Wide bore gradient coil system representing clinical systems with average length static magnet designs. | 70 | 150 |

**Table S2**. Characteristics of Virtual Population human body models used in simulations.

| **Virtual Population Model Name** | **Model  acronym** | **Gender** | **Height (m)** | **Mass (kg)** | **BMI (kg/m^2^)** |
| --- | --- | --- | --- | --- | --- |
| Duke | DUKE | male | 1.77 | 70.2 | 22.4 |
| Ella | ELLA | female | 1.63 | 58.7 | 22.1 |
| Ella (morphed) | EL26 | female | 1.63 | 69.4 | 26.1 |
| Ella (morphed) | EL30 | female | 1.63 | 79.7 | 30.0 |
| Fats | FATS | male | 1.82 | 119.6 | 36.1 |
| Glenn | GLEN | male | 1.73 | 65.0 | 21.7 |

## Feature Selection


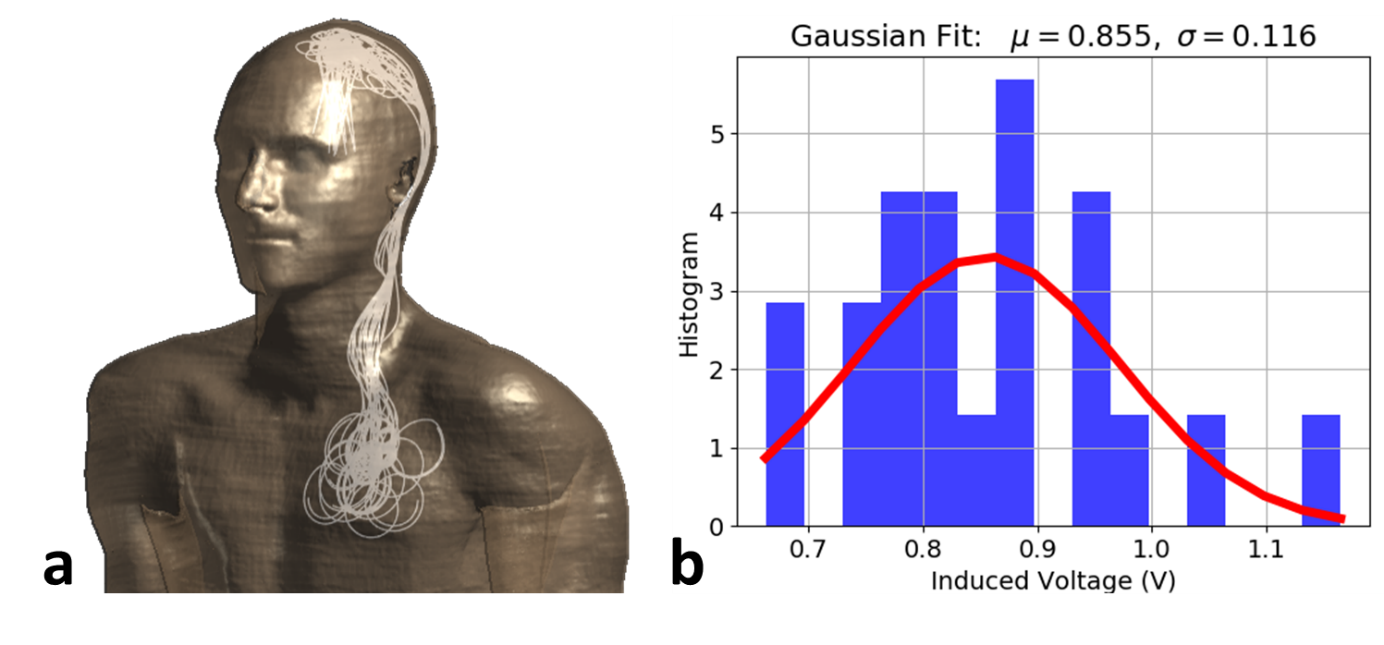


Figure S2. (a) Replicate DBS routing pathways with a 40 cm long lead implanted in left brain hemisphere, connected to a 60 cm extension that is tunneled through the left side of the neck and connected to a neurostimulator implanted in the pectoral region are shown. Selected feature set cannot uniquely identify individual routing pathways. (b) Histogram of induced-voltage values of replicate routing pathways and a Gaussian fit to the histogram are shown. Full-Dataset includes each unique routing pathway (all values in the histogram), whereas Condensed-Dataset includes average gradient-induced voltage value (e.g. mean = 0.855 V) of these replicate routing pathways.

# Results

## Data Exploration


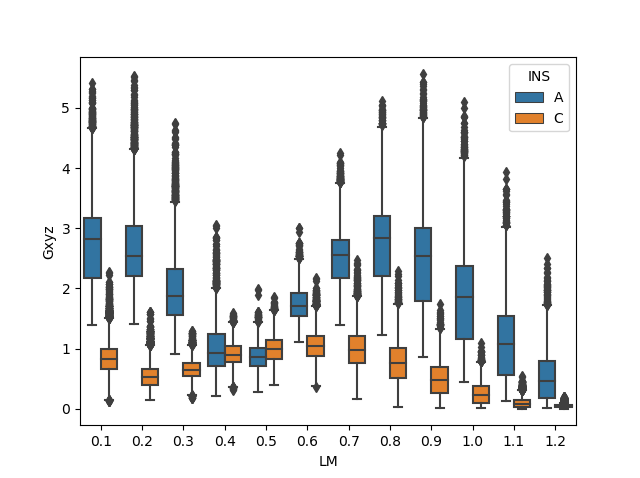


Figure S3. Gradient induced voltage boxplot with x-axis representing scan landmark location. Blue and orange traces represent abdominal and pectoral INS implant locations, respectively.


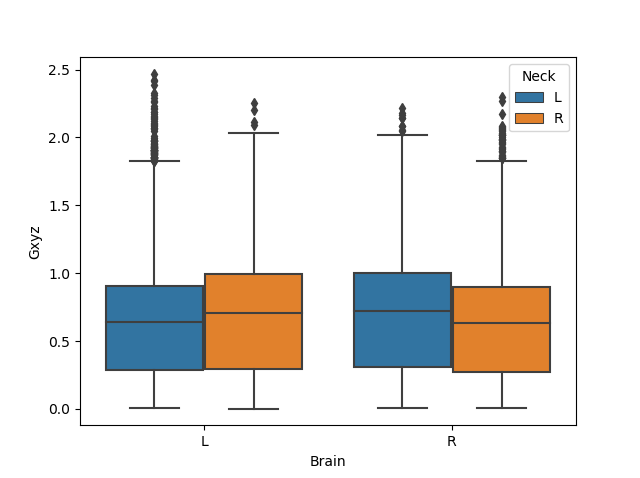


Figure S4. Gradient induced voltage boxplots with x-axis representing brain hemisphere DBS lead is implanted and hue represent side of the neck DBS extension is tunneled. On average, contralateral routing pathways have slightly higher induced voltage levels.

## Machine Learning Algorithm Selection


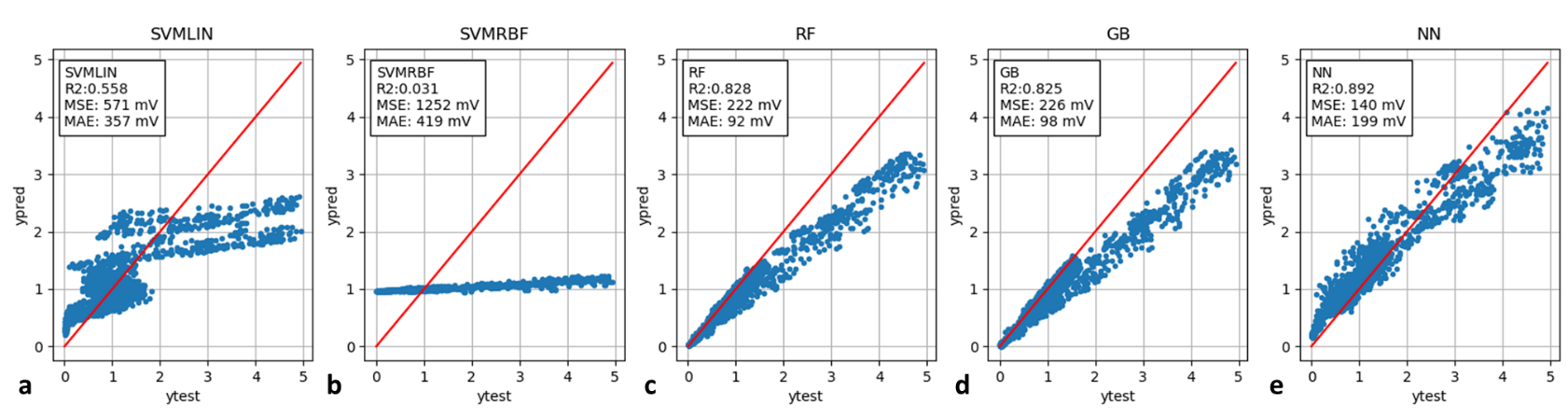


Figure S5. Performance metrics of (a) support vector regressor with linear kernel (SVMLIN), (b) support vector regressor with radial basis functions (SVMRBF), (c) random forest regressor, (d) gradient boosting regressor and (e) fully-connected artificial neural network with two hidden layers are plotted. Predictive models are trained using five anatomical models excluding “Fats”, and performance are evaluated on the “Fats” induced voltage dataset. R2, mean-squared error (MSE) and median-absolute error (MAE) values are annotated inside sub-figures.

## Neural Network Performance


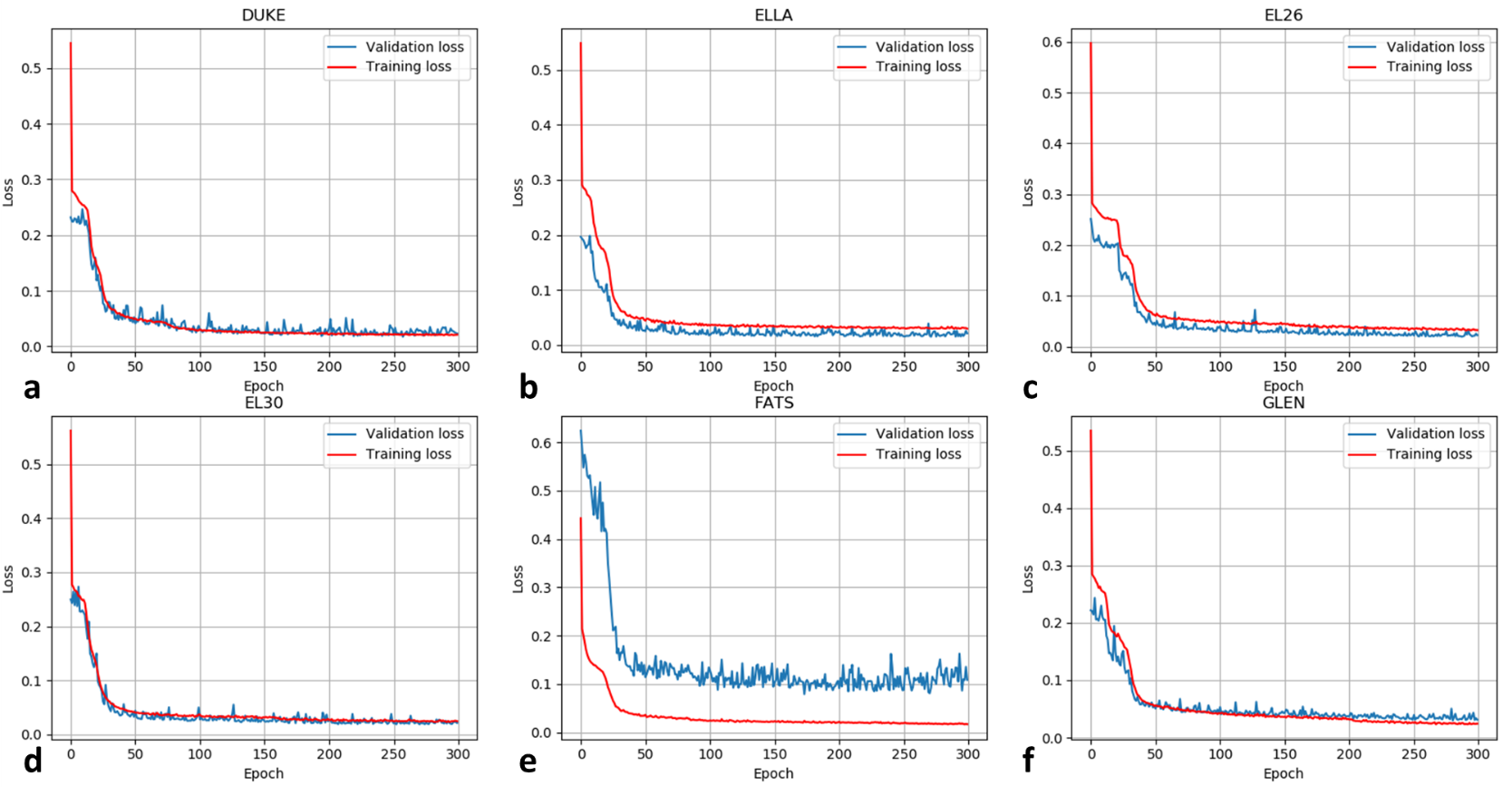


Figure S6. Neural network leave-one-out model training history (red: mean-squared-error training loss, blue: mean-squared-error validation loss) are plotted. Body-model annotations indicate the model left out from training set and the body-model in the validation set.
